# Impulsive time series modeling with application to luteinizing hormone data

**DOI:** 10.3389/fendo.2022.957993

**Published:** 2022-11-01

**Authors:** Håkan Runvik, Alexander Medvedev

**Affiliations:** Department of Information Technology, Uppsala University, Uppsala, Sweden

**Keywords:** endocrine regulation, impulsive hormone secretion, mathematical modeling, luteinizing hormone, gonadotropin-releasing hormone, ganirelix, cortisol, adrenocorticotropic hormone

## Abstract

This work considers the estimation of impulsive time series pertaining to biomedical systems and, in particular, to endocrine ones. We assume a signal model in the form of the output of a continuous linear time-invariant system driven by a sequence of instantaneous impulses, which concept is utilized here, in particular, for modeling of the male reproductive hormone axis. An estimation method to identify the impulsive sequence and the continuous system dynamics from sampled measurements of the output is proposed. Hinging on thorough mathematical analysis, the method improves upon a previously developed least-squares algorithm by resolving the trade-off between model fit and input sparsity, thus removing the need for manual tuning of user-defined estimation algorithm parameters. Experiments with synthetic data and Markov chain Monte-Carlo estimation demonstrate the viability of the proposed method, but also indicate that measurement noise renders the estimation problem ill-posed, as multiple estimates along a curve in the parameter space yield similar fits to data. The method is furthermore applied to clinical luteinizing hormone data collected from healthy males and, for comparability, one female, with similar results. Comparison between the estimated and theoretical elimination rates, as well as simulation of the estimated models, demonstrate the efficacy of the method. The sensitivity of the impulse distribution to the estimated elimination rates is investigated on a subject-specific data subset, revealing that the input sequence and elimination rate estimates can be interdependent. The dose-dependent effect of a selective gonadotropin releasing hormone receptor antagonist on the frequency and weights of the estimated impulses is also analyzed; a significant impact of the medication on the impulse weights is confirmed. To demonstrate the feasibility of the estimation approach for other hormones with pulsatile secretion, the modeling of cortisol data sets collected from three female adolescents was performed.

## 1 Introduction

Despite the critical role of the endocrine system in the normal functioning of the organism, it remains an open question how to mathematically model the process of hormone release and interaction in a parsimonious way and yet explain experimentally observed behaviors. Two challenges in this regard are the complex interactions involved and the limitations regarding sampling frequency, experiment length, and total amount of blood drawn from a subject in clinical studies. One key feature that is required in endocrine models is the representation of both continuous hormone release and pulsatile concentration bursts, as a majority of hormone secretion patterns display pulsatility (1). It is therefore judicious to use hybrid models, i.e., models that combine continuous and discrete dynamics in the system description.

The hybrid model and the corresponding estimation problems that we investigate below, originate from the male testosterone regulation model introduced in (2, 3). Three main hormones are involved in this regulation: gonadotropin-releasing hormone (GnRH), luteinizing hormone (LH), and testosterone (Te). In the present work, the estimation of the pulsatile release of GnRH and the elimination rates of GnRH and LH from sampled serum measurements of LH are considered in detail. Modeling of cortisol is also performed, but without much subsequent analysis as only limited data have been made available to the authors. We term the considered signal model, where an unknown sequence of impulses drives a continuous linear time-invariant system of a given structure but with unknown parameters, an *impulsive time series*. The resulting estimation problem is, however, of a general nature and applications both to cortisol regulation (4) and to pharmacokinetics (5) have been considered in the past.

The conventional approach to pulsatile endocrine data analysis can be briefly summarized as follows. The hormone concentration *C*(*t*) with the initial condition *C*(0) is modeled as the convolution of the secretion rate *S*(*t*) and the impulse response of the system *E*(*t*) describing the hormone elimination rate


(1) $$C\left(t\right)=\int_{0}^{t}S\left(\tau \right)E\left(t-\tau \right)d\tau +C\left(0\right)E\left(t\right).$$

A number of techniques have been proposed for recovering the input signal or/and the parameters for this type of system. In an experimental data set, only measurements of *C*(*t*) are typically available, with the rest of the involved quantities being more or less unknown. A classical approach to resolving this issue, termed deconvolution, is to assume the function *E*(*t*) known and estimate the secretion profile *S*(*t*) as the input of a dynamical system. An early work covering a comparison of three linear methods, namely least-squares deconvolution, Maximum a Posteriori (MAP) deconvolution, and Wiener deconvolution, is found in DeNicoalo, 1993 (6). The linear approaches are known to exhibit high sensitivity of the estimate to modeling uncertainty and signal uncertainty, i.e., disturbance. This is due to the fact that the deconvolution operation attempts to invert the system dynamics, which operation is inherently ill-conditioned.

To yield biologically meaningful estimates, assumptions on the signal shape of *S*(*t*) are required. For instance, a Gaussian-shaped input is assumed in (7) with the deconvolution performed using nonlinear least squares, given prior information on the approximate location of the pulsatile secretion episodes. Another popular option accounting for the experimentally observed skewed shape of pulsatile hormone concentration excursions is a Gamma function shaped input, e.g (8). Numerous improvements to the deconvolution-based estimation algorithms have been suggested since then, including the Bayesian setup in (9) and the automated deconvolution algorithm presented in (10).

Software packages supporting hormone data deconvolution analysis are publicly available. The most widespread and validated software is AutoDecon described in (10). AutoDecon employs a further development of the Cluster algorithm presented in (11) and estimates hormone half-life time, basal secretion, and initial hormone concentration. Another example is WINSTODEC (12), based on stochastic deconvolution.

It is, however, important to note that even with disturbance-free measurement data, the joint impulse and time-constant estimation problem is ill-posed since there are always multiple solutions. The reason for this is that the faster dynamics of the linear system can be compensated for by introducing more input impulses. However, as shown in (13), a unique solution can be identified under the assumption that the impulses of the generating process are sufficiently sparse. Under uncertainty, the ill-posedness necessitates a trade-off between model fit and impulse sparsity. To resolve this issue, either regularization or statistical tools such as cross-validation or information criteria can be used. The *ℓ*_1_ -regularized least-squares formulation in (14) is an example of the former, while a statistical test based on variance-of-fit is employed in (10). In (4), both regularization and cross-validation are used. For Bayesian algorithms, regularization can instead be implemented through the choice of prior distributions (but, as we will note later in this work, it is not clear whether regularization is as important in such a setup).

Many of the algorithms mentioned above rely on tuning parameters or heuristics to determine the degree of estimate regularization. Instead, we employ here a method that was proposed in (13), where the correct trade-off between fit and sparsity is derived mathematically and included in the estimation algorithm. It is based on the least-squares method from (14), but instead of the regularized formulation that is combined with gridding in that work, a parameter-dependent optimization formulation is used. It utilizes the way in which the residual sum of squares depends on the parameters of the linear system, so that the time constants can be determined without the need for user-defined regularization.

In addition to the trade-off between impulse sparsity and model fit, a second type of ill-posedness is identified in this work. It is linked to the well-researched problem of estimating sums of exponential functions (see e.g (15), for an overview of the topic), and manifests itself as a functional relation between the parameters of the linear plant, i.e., a curve in the corresponding plane. A subset of the parameter estimates located along this curve typically give very similar fits to the data. This problem type is also well-known in pharmacokinetic models, but the particular relationship highlighted here has, to the best of the knowledge of the authors, not been discussed previously.

The rest of this paper is organized as follows. First, the model, estimation problem, and the least-squares estimation method are presented, along with a Markov chain Monte-Carlo (MCMC) method. The latter is used in the following section, to demonstrate the validity of the approach on synthetic data. Further, estimation was performed on clinical LH data, investigating the distribution of time constants between individuals as well as the dose-dependent effect of a GnRH-receptor antagonist on impulse weights and amplitudes. Finally, estimation is performed on three data sets constituting cortisol data to illustrate an application to other endocrine axes.

## 2 Materials and methods

The model at hand includes GnRH, which is released in a pulsatile manner, and LH, whose secretion is stimulated by the GnRH concentration. The LH concentration can be measured in blood samples, while GnRH cannot be directly measured in humans for ethical reasons. Under the idealized assumption that the GnRH-bursts correspond to instantaneous releases of the hormone into the bloodstream, the bursts can be portrayed by a sequence of weighted Dirac impulses. Then the firing times of the impulses mark the GnRH release events, while the impulse weights represent the secreted hormone amounts. Assuming linear elimination, the system can be expressed in state space form as


(2) $$\dot{x}=Ax+B\xi \left(t\right),\;y=Cx,$$

where


, $$x=\left[x_{1}x_{2}\right],\;A=\left[-b_{1}g_{1}-b_{2}0\right],\;B=\left[10\right],\;C={\left[01\right]}^{⊤},$$

*x*_1_ is the concentration of GnRH, *x*_2_ is the concentration of LH, *b*_1_ and *b*_2_ are the (positive) time constants of their eliminations, and *g*_1_ is a positive parameter that describes the stimulation of LH production by GnRH. The input signal is given by


(3) $$\xi \left(t\right)=\sum_{n=0}^{\infty }d_{n}\delta \left(t-\tau_{n}\right),$$

where *δ*(·) is the Dirac delta function and *d*_*n*_ and *τ*_*n*_ determine the positive impulse weights and times. The output signal of this system is evaluated to


$$y\left(t\right)=C\left({\text{e}}^{A\left(t-t_{0}\right)}x_{0}+\int_{t_{0}}^{t}{\text{e}}^{A\left(t-\tau \right)}B\xi \left(\tau \right)\text{ d}\tau \right)$$

(4) $$=C{\text{e}}^{A\left(t-t_{0}\right)}x_{0}+\sum_{n=0}^{\infty }g_{1}d_{n}z\left(b_{1},b_{2},t-\tau_{n}\right),$$

where


$$z\left(b_{1},b_{2},t\right)=\frac{e^{-b_{2}t}-e^{-b_{1}t}}{b_{1}-b_{2}}H\left(t\right),$$

and *H*(*t*) is the Heaviside step function. The output *y*(*t*) of Equation (4) is then termed an impulsive time series. It is a time series since the signal *ξ*(*t*) is not available for measurement and the time series is impulsive since *ξ*(*t*) consists of instantaneous impulses.

Assuming the model structure above, the impulse times and weights as well as the linear elimination rates are to be estimated from measurements of the output *y*(*t*) sampled at times *t*_*k*_, where *k*=1,…,*K* and *t*_*k*_<*t*_*k*+1_. Notice also that *ξ*(*t*) is a theoretical construct and cannot be captured by sampling. Although this problem is presented here in the context of the male reproductive hormone axis, the general character of the linear plant and the input signal permits application to other biomedical systems.

Two assumptions are needed to secure the feasibility of the formulated estimation problem. First, the parameter *g*_1_ cannot be uniquely identified from the output and is, without loss of generality, set *g*_1_=1. This can also be seen as a normalization of the weights *d*_*n*_ . Second, we also restrict the estimates of the impulse times to coincide with the sampling times of the measurements of *y*(*t*). Since impulse times in between sampling instances cannot be identified, as proved in (14), this is not a restrictive assumption, but it rather reflects an inherent ambiguity in the estimation problem, which can only be resolved by more frequent sampling, see (16). Notice also that model (4) is defined for *t* ∈ [0,*∞*) whereas only a finite data set is available in practice. Therefore, the actual number of *δ* -functions captured in the data set is unknown. By allowing one impulse at each sampling time of the data set and selecting proper weights *d*_*n*_, a solution to the estimation problem yielding a zero output estimation error can be obtained. Thus, in contrast with stochastic time series analysis, the output estimation error cannot be considered here as the sole model performance criterion.

Two distinct methods were employed to solve the estimation problem at hand. The first one, which is covered in *Least squares estimation*, is based on a least-squares setup formulated in (14). The second one, presented in *Adaptive Metropolis estimation*, utilizes a probabilistic approach where Markov Chain Monte-Carlo (MCMC) estimation is used to derive posterior probability distributions for the parameters. The latter method will in particular be exploited to investigate the functional relation between the estimated time constants of the system that which is predicted by the former.

### 2.1 Least-squares estimation

To obtain a least squares formulation of the estimation problem, introduce the measurement vector


$$Y={\left[\begin{array}{ccc} y\left(t_{1}\right) & \ldots & y\left(t_{K}\right) \end{array}\right]}^{⊤},$$

which, using Equation (4) for the output, enables the matrix formulation


$$Y=\text{Φ}\left(b_{1},b_{2}\right)\theta .$$

Here


$$\text{Φ}\left(b_{1},b_{2}\right)={\left[\begin{array}{ccc} \varphi \left(b_{1},b_{2},t_{1}\right) & \ldots & \varphi \left(b_{1},b_{2},t_{K}\right) \end{array}\right]}^{⊤},$$

where


$$\varphi \left(b_{1},b_{2},t_{i}\right)=\left[\begin{array}{c} {\text{e}}^{b_{2}\left(t_{i}-t_{1}\right)}\\ z\left(b_{1},b_{2},t_{i}-t_{1}\right)\\ \vdots \\ z\left(b_{1},b_{2},t_{i}-t_{K-1}\right) \end{array}\right],$$

and


$$\theta ={\left[\begin{array}{cccc} x_{2}\left(t_{1}\right) & d_{1} & \ldots & d_{K-1} \end{array}\right]}^{⊤}.$$

Notice that the impulse and sampling times, as specified before, are assumed to coincide, and that in this discretized formulation, zero impulse weights are permitted, as opposed to the expression in Equation (3) for the input. The initial state of the system is encoded in the first two elements of *θ* (a nonzero *x*_1_(*t*_1_) can equivalently be represented by an impulse).

We thus aim to estimate the parameters *b*_1_,*b*_2_ and the vector *θ* from the measurements in *Y*, which are typically corrupted by noise. However, with unknown parameters in both Φ (*b*_1_,*b*_2_) and *θ*, ordinary least squares cannot alone be used to perform the estimation. In particular, for sufficiently high values of *b*_1_ and *b*_2_ , a perfect match to an arbitrary data set can be obtained with positive impulse weights *d*_*i*_ at every sampling instant. However, this does not correspond to a physiologically relevant or practically useful solution. In view of these challenges, the problem will be recast as a parameter-dependent optimization problem, *via* the formulation


(5) $$\hat{\theta }\left(b_{1},b_{2}\right)=\underset{\theta }{\arg\min}\|Y-\text{Φ}\left(b_{1},b_{2}\right)\theta {\|}^{2},$$

where ‖·‖ is the Euclidean vector norm and the values of *b*_1_,*b*_2_ are determined in an outer level optimization. We will present the key parts of this method here, with further details provided in **Supplementary Material**. The full derivation can be found in (13).

#### 2.1.1. Outer level optimization

**Figure 1:**
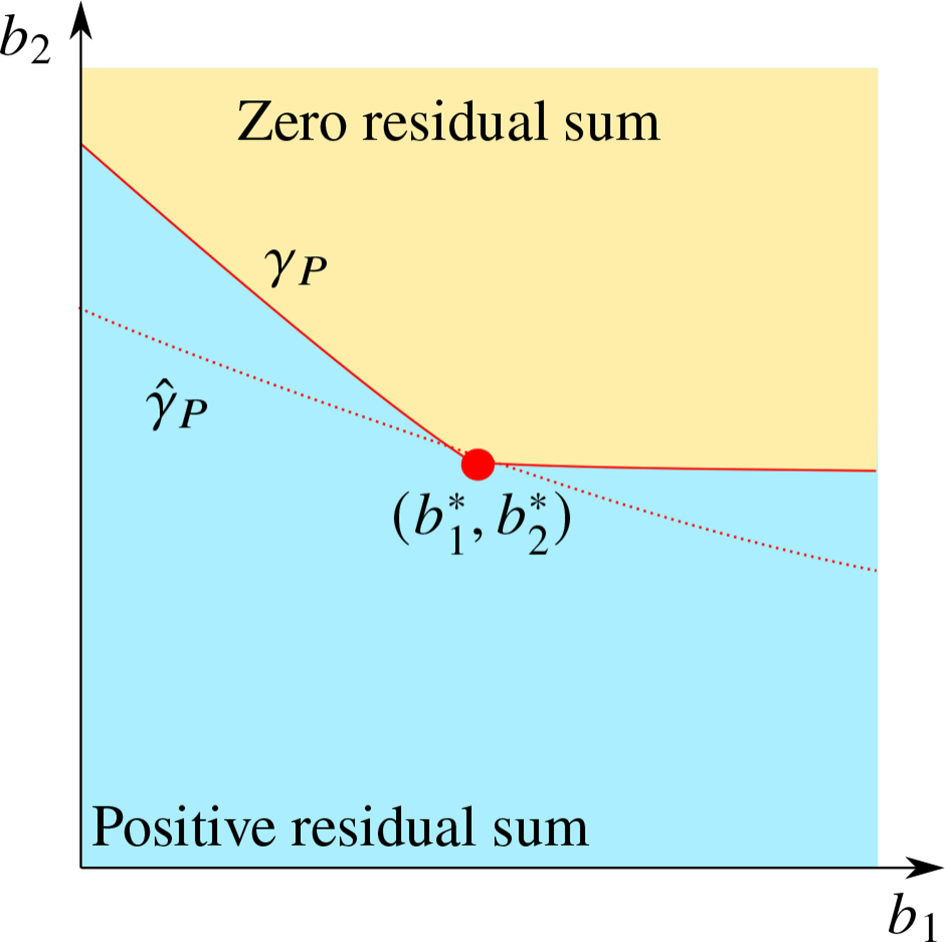
Illustration of the qualitative behavior of the curve *γ*_*P*_ (red solid line) and its estimate ${\hat{\gamma }}_{P}$ (red dotted line) in the *b*_1_ - *b*_2_ -parameter space, in relation to the true parameter values $\left(b_{1}^{*},b_{2}^{*}\right)$ (red dot).

In the outer level optimization, noise-corrupted measurements generated from system (2) with $b_{1}=b_{1}^{*},b_{2}=b_{2}^{*}$ and the impulse weights $d_{k}^{*}$ are assumed, i.e.,


$$Y=\text{Φ}\left(b_{1}^{*},b_{2}^{*}\right)\theta^{*}+\varepsilon ,$$

where


$$\theta^{*}={\left[\begin{array}{cccc} x_{2}^{*}\left(t_{1}\right) & d_{1}^{*} & \ldots & d_{K-1}^{*} \end{array}\right]}^{⊤},$$

and *ε* is a zero-mean noise vector. The goal of the optimization is to find estimates of $b_{1}^{*},b_{2}^{*}$ . As it will be shown later, this goal is often not achievable in practice, and the estimation of a curve *γ*_*P*_ in the *b*_1_ - *b*_2_ -plane will be the focus of this section. The curve *γ*_*P*_ is defined as the boundary of the region where, if noise-free measurements were available, non-negatively constrained impulses would give a perfect fit (zero loss) to the data for the optimization problem in Equation (5). In particular, as shown in **Figure 1**, the point $\left(b_{1}^{*},b_{2}^{*}\right)$ always belongs to *γ*_*P*_ since the measurements are generated by positively weighted impulses, but, if the values of *b*_1_ or *b*_2_ are decreased, the residual sum of squares becomes non-zero.

Introduce the residual error *g*(*b*_1_,*b*_2_) , i.e.,


$$g\left(b_{1},b_{2}\right)=\|Y-\text{Ф}\left(b_{1},b_{2}\right)\hat{\theta }\left(b_{1},b_{2}\right){\|}^{2}.$$

Under measurement noise or model uncertainty, an estimate ${\hat{\gamma }}_{P}$ of the sought curve can be found by approximating *g*(*b*_1_,*b*_2_) as a quadratic function of the form given in the lemma below, when


$$b_{1}={\overset{ˉ}{b}}_{1},\;b_{2}<{\overset{ˉ}{b}}_{2},$$

and where $\left({\overset{ˉ}{b}}_{1},{\overset{ˉ}{b}}_{2}\right)\in \gamma_{P}$ .

**Lemma 1.** (13) *Let f*(*x*)=*c*_1_(*x*−*x* ^*^)^2^+*c*_2_, *where c*_1_,*c*_2_>0. *Define N*_*f*_(*x*) and $\hat{x}$ by


$$N_{f}\left(x\right)=-\frac{f\left(x\right)}{df\left(x\right)/dx},$$

x^=argminx Nf(x)+minx Nf(x),

s.t.minx Nf(x)>0.

Then $\hat{x}=x^{*}$ .

**Figure 2:**
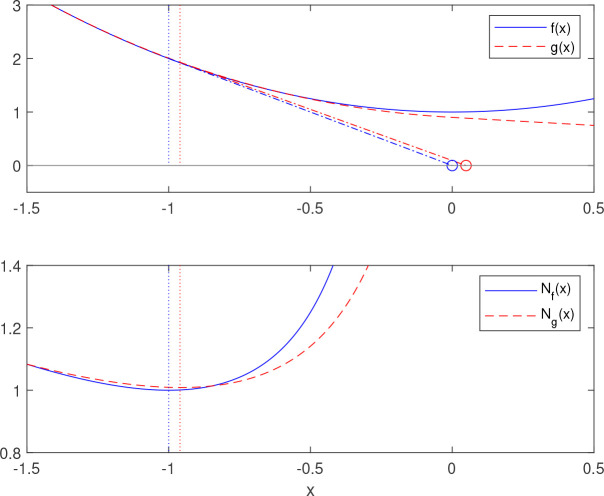
Minimization of a quadratic function according to Lemma 1. The function *f*(*x*) is quadratic and the corresponding *N*_*f*_(*x*) in the lower plot attains the minimal value 1 for *x*=−1 (blue dotted line). In the upper plot, the intersection of the tangent of *f*(−1) and the *X*-axis corresponds to the minimizer *X*=0. The function *g*(*x*) is an approximation of *f*(*x*) . By minimizing *N*_*g*_(*x*) (red dotted line) and drawing the tangent in the same way, an approximation of the minimized of *f*(*x*) is obtained.

Note that *N*_*f*_(*x*) corresponds to a step in Newton’s root finding method and that the above construction is equivalent to letting $\hat{x}$ be the intersection between the tangent line of $f\left(\tilde{x}\right)$ and the *x* -axis, where $\tilde{x}$ is the point where the step size is minimized, see **Figure 2**.

We therefore define


$$N_{g}\left(b_{1},b_{2}\right)=-\frac{g\left(b_{1},b_{2}\right)}{\partial g\left(b_{1},b_{2}\right)/\partial b_{2}},$$

and formulate the estimation of a point $\left({\hat{b}}_{2}\left(b_{1}\right),b_{1}\right)\in {\hat{\gamma }}_{P}$ as


(6) b˜2(b1)=argminb2 Ng(b1,b2),s.t.#{dk>dΠ}≤Π

(7) $${\hat{b}}_{2}\left(b_{1}\right)={\tilde{b}}_{2}\left(b_{1}\right)+N_{g}\left(b_{1},{\tilde{b}}_{2}\left(b_{1}\right)\right),$$

where # denotes set cardinality and *d*_Π_>0 , Π∈*ℕ* are chosen to prohibit solutions with redundant input impulses. This corresponds to the condition on *min* *N*_*f*_(*x*) in Lemma 1. We thus assume that the residual sum is quadratic in*b*_2_ below *γ*_*P*_, See **Figure 1**. Note that this is not the case above *γ*_*P*_ as the faster dynamics then permits more impulses and, therefore, a better fit to the data. We then utilize Lemma 1 that permits the minimization of such a function without actually evaluating the function close to the minimum. This is illustrated in **Figure 2**, where *g*(*x*) can be used to approximate the minimum of *f*(*x*) even though *g*(*x*) itself is monotonous, which means the residual sum is expected to be with respect to both *b*_1_ and *b*_2_.

A typical qualitative behavior of the estimate ${\hat{\gamma }}_{P}$ , observed in numerical experiments, is indicated in **Figure 1**; the shape of *γ*_*P*_ is not reproduced, but the distance between ${\hat{\gamma }}_{P}$ and the point $\left(b_{1}^{*},b_{2}^{*}\right)$ is small.

In (13), an optimization formulation over both *b*_1_ and *b*_2_ was also presented to estimate $b_{1}^{*}$ and $b_{2}^{*}$ directly, rather than *γ*_*P*_. However, this method is only useful when the noise level is quite low, and will therefore not be considered here, as an application to clinical data is foreseen.

#### 2.1.2. Estimation algorithm

The main part of the estimation algorithm consists of the estimation of the curve *γ*_*P*_ . We therefore suggest gridding over a suitable range of values for *b*_1_ and solving the optimization problem in Equation (6) at each point to obtain an estimate of the curve using Equation (7). Since this optimization problem is not necessarily convex, we use gridding in the *b*_2_-direction to find the minimum in all experiments, except for the setup with different levels of administered GnRH-receptor antagonist described in *Effects of GnRH-receptor antagonist and age*. The estimation results from the data set of each unmedicated individual are used to initiate a local optimization that is utilized in the cases when medication is administered. To obtain impulse estimates, optimization problem (5) is then solved along the curve, after the removal of impulses with weights below a threshold *d*_min_>0. In the present work, this parameter is chosen as a few percent of the maximal estimated impulse weight. In general, a smaller value of *d*_*min* _ is suitable when the noise level is lower, since the risk of misattributing noise impact to the effect of small impulses is then lower. Adjacent impulses are then merged according to the formula in (14). The steps of the estimation are summarized in Algorithm 4.1.2, with further details provided in **Supplementary Material**.

**Algorithm 1:** Impulse and time constant estimation.

1: Create grid ${\left\{b_{1}^{\left(n\right)}\right\}}_{n=1,\ldots ,N}$
2: **for** *n*ϵ{1,…,*N*}**do**
3: Let $b_{1}=b_{1}^{\left(n\right)}$
4: Calculate ${\hat{b}}_{2}\left(b_{1}\right)$ using (6) and (7)
5: Calculate $\hat{\theta }\left(b_{1},{\hat{b}}_{2}\left(b_{1}\right)\right)$ from (5)
6: Let $S=\{kЄ\left\{1,\ldots ,K\right\}|{\hat{d}}_{k}<d_{\text{min}}\}$
7: Solve (5) with all *d*_*k*_ with *k*ϵ*S* constrained to be zero
8: Merge adjacent non-zero impulses according to Algorithm 1 in (14)
9: **end for**

One may note that even though this algorithm does rely on regularization in the sense that impulses below a given threshold are removed, and that for this regularization, another approach, such as *ℓ*_1_-regularization, could be potentially preferable. However, there is an important distinction between obtaining a sparse input sequence when the time constants are fixed, compared to deciding the trade-off between sparsity and model fit with free time constants. In the former case, the characteristics of the simulated output of the estimated system are largely unaffected by the removal of impulses, so changing the impulse threshold does not result in significant quantitative changes in the solution. In the experimental setup described in *Functional relation between the elimination rates*, for example, most of the estimated impulse weights are smaller than 1% of the maximal estimated impulse weight. In the latter case, on the contrary, the characteristics of the solution could change significantly depending on the regularization. For example, as the value of the regularization parameter *λ*_max_ in (14) is increased, the solution converges towards faster dynamics and impulse weights that are significantly greater than zero at every sampling time.

### 2.2. Adaptive Metropolis estimation

The estimates resulting from the method presented in the preceding section will be compared against posterior parameter distributions obtained with the adaptive Metropolis (AM) algorithm (17). A brief description of Markov Chain Monte-Carlo (MCMC) and the AM algorithm will be given here. For a more comprehensive exposition, see e.g (18), and (17).

The AM algorithm is a version of the Metropolis–Hastings algorithm (19), which is an MCMC method. Here we use it to generate samples from the posterior distributions of the parameters of the system, i.e., the parameters *τ*_*n*_, *d*_*n*_, *b*_1_, and *b*_2_ are treated as random variables whose joint posterior probability distribution is sought. In contrast with the least-squares algorithm, the MCMC setup does not rely on an explicit formulation of the optimization problem. Instead, the forward model, i.e (4), is used together with the calculation of a likelihood function. In particular, it is not assumed that the impulses occur at the sampling times. With the uncertainty modeled as additive Gaussian noise, the logarithmic likelihood can be explicitly calculated from the residual sum of squares. We furthermore assume uniform priors, making the posterior probability distribution proportional to the likelihood. The AM algorithm, which samples this distribution through a random walk in the parameter space, is characterized by the use of an empirical covariance matrix to scale the proposal distributions, i.e., the proposed steps of the random walk.

A challenge with performing MCMC estimation for the problem at hand is the unknown number of impulses, which means that the dimension of the parameter space is unknown. In (9), a birth-death MCMC method was employed to solve this problem [see also the reversible jump MCMC developed in (20)]. Here we use an algorithmically simpler approach of performing the estimation with a few more impulses than expected to be needed (in our experiments, five rather than three) and then removing or merging superfluous impulses from the generated samples of the posterior distribution in a post-processing step. For synthetic data, it is of course also possible to use the true number of impulses for the AM algorithm, but, even in this case, a higher number can be beneficial, as it reduces the risk of impulses being missed due to the algorithm being stuck at local posterior distribution maxima. The superfluous impulses are identified by their low weight, being close to the end of the estimation time horizon, or being so close to a neighboring impulse that they can be merged with minimal or no effect on the output at the sampling times using the technique proposed in (14). The convergence of the Markov chain is then evaluated using the Gelman–Rubin statistic (21) for the reduced parameter set. More details on the MCMC estimation are provided in the **Supplementary Material**.

The adopted approach relies on the MCMC setup providing sparsity implicitly, i.e., that proposals with a larger number of significant impulses than the generating model are unlikely to be accepted. This is in contrast to the least-squares approach, where, as shown by the analysis in *Least squares estimation*, explicit measures are needed to obtain a sparse solution. An intuitive explanation for the inherent sparsity of the MCMC estimation is that solutions with many impulses, which could result in a better fit for noisy data, correspond to very narrow peaks in the probability distribution, which makes them unlikely to be sampled. However, a formal analysis of this hypothesis has not been performed.

## 3 Results

Experimental results with synthetic and clinical data are presented in this section. More details on the specific estimation setups used in each experiment are provided in the **Supplementary Material**.

### 3.1. Functional relation between the elimination rates

**Figure 3:**
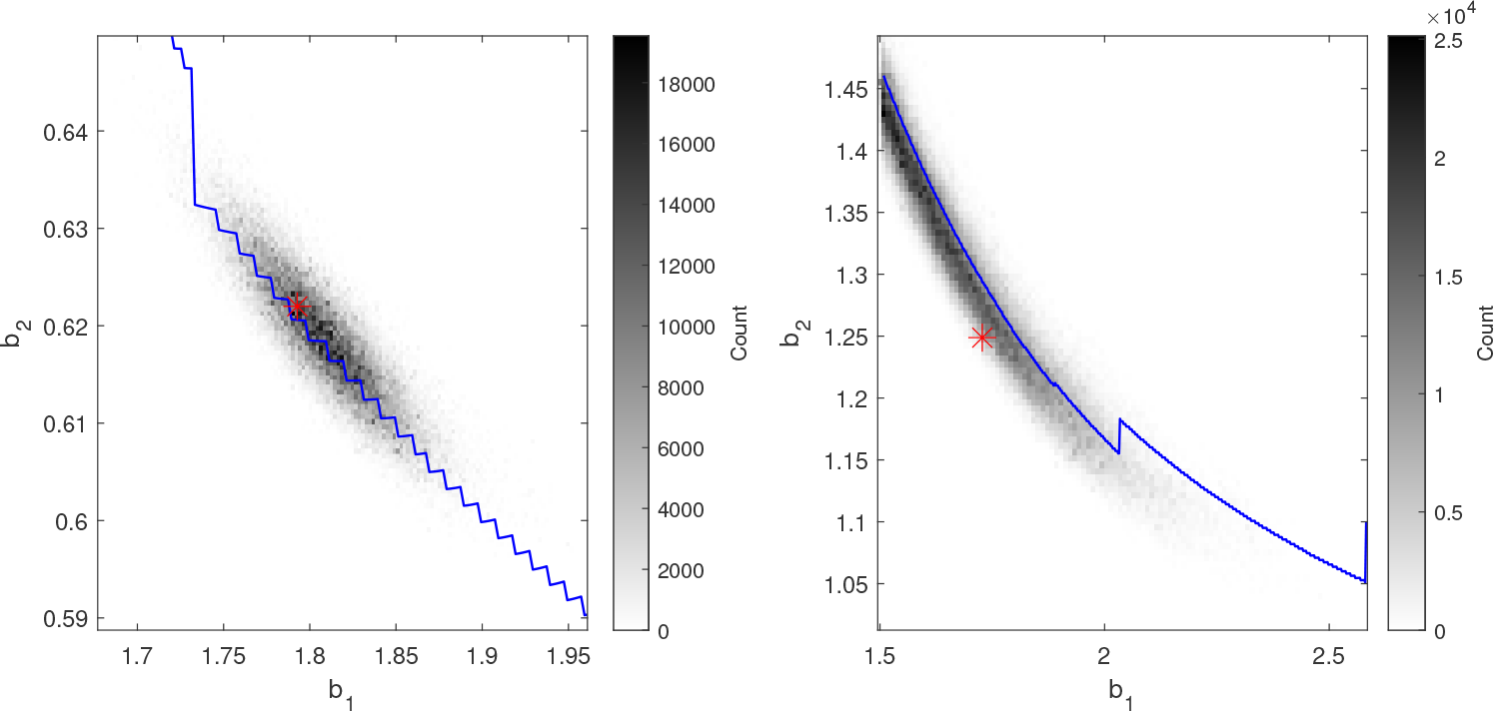
Histograms of the posterior distribution of *b*_1_ and *b_2_* from the AM algorithm (gray-scale) together with the estimated ${\hat{\gamma }}_{P}$ (blue curves) and the generating parameter values (red asterisks) for two synthetic data sets. The ${\hat{\gamma }}_{P}$ -curves coincide with ridges in the posterior distributions and the minimal distances between the curves and the generating parameters are short.

**Figure 4:**
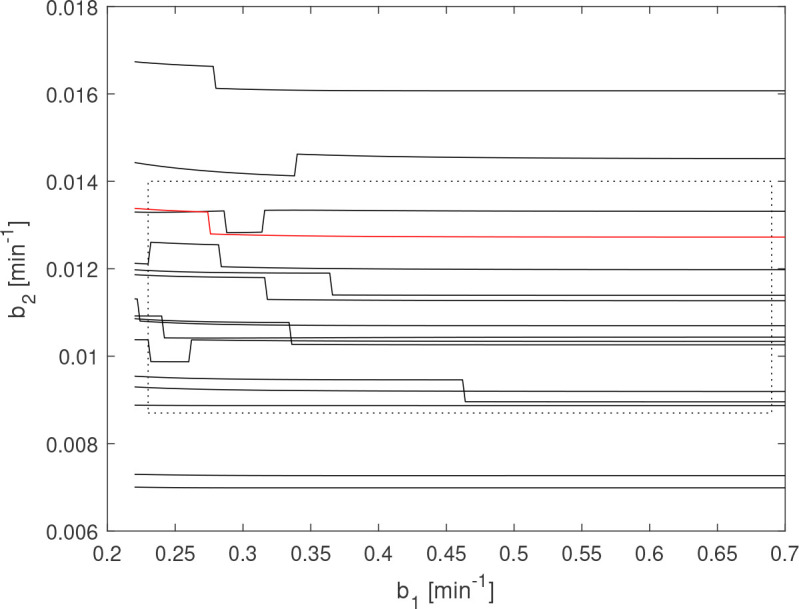
Estimated *γ*_*P*_ for LH-data from 15 healthy males (solid black lines), one female (solid red) and the theoretical parameter ranges (dotted box) given in (22).

The derivation of the least-squares algorithm indicated that the estimation problem could be ill-posed in the presence of noise, in the sense that multiple (*b*_1_,*b*_2_) -pairs along the ${\hat{\gamma }}_{P}$ -curve give similar fits to the data. To examine this hypothesis, the estimated curve obtained from the least-squares method is compared with the posterior distributions obtained using the AM algorithm. Synthetic data were used in this experiment; the parameters used to generate the data are given in the **Supplementary Material**. Note that the synthetic data do not mimic LH measurements; the elimination rates are instead chosen to be more similar in magnitude to those of GnRH and LH. With a large discrepancy between the elimination rates, it is clear that the estimation uncertainty will mostly be in the direction of the faster rate, while the parameter ranges used here permit significant uncertainty in both parameters (compare **Figures 3**, **4**). This makes the setup more suitable for highlighting the capabilities of the estimation algorithm.

For each data set, four chains of length 3*e*6 are generated with the AM algorithm, with a burn-in of 1*e*6 . The results are then merged to give an approximation of the posterior distribution. Using the Parallel Computing Toolbox in Matlab, the MCMC estimation for each data set takes approximately 5 to 10 min on a standard laptop with four 1.9 GHz cores. The estimate ${\hat{\gamma }}_{P}$ obtained from the least-squares algorithm, on the other hand, takes approximately 40 s per data set to compute. One should, however, note that no particular optimization of the code in terms of performance has been performed.

To ensure that only converged chains are included in the analysis of the results, a simple strategy of discarding data sets where the mean Gelman-Rubin statistic exceeds 1.2 is employed. This might introduce a bias in the results, but since the number of rejected data sets is low and the AM algorithm is only used for comparison, this problem is deemed negligible in the current context.

#### 3.1.1. Experimental results

In **Figure 3**, the marginal posterior distribution in the *b*_1_ - *b*_2_-plane is illustrated with histograms together with the estimate ${\hat{\gamma }}_{P}$ for two runs. The probability distributions are consistent with the least-squares estimates as the ridges which represent the highest densities approximately coincide with the ${\hat{\gamma }}_{P}$ -curves. However, the regions of highest density do not necessarily coincide with the true parameter values. This is particularly evident for the plot to the right. It therefore appears as the ${\hat{\gamma }}_{P}$ defines a direction along which the uncertainty of the parameter estimates is high.

To formalize the analysis, we introduce the set Ω_*P*_ with ${\hat{\gamma }}_{P}$ as its boundary:


$${\text{Ω}}_{P}=\{\left(x,y\right)\in {\mathbb{R}}^{2}|\exists z\in \mathbb{R}\text{ s}.\text{t}.\left(x,z\right)\in {\hat{\gamma }}_{P}\text{ and }z<y\}.$$

For each sample of the posterior, the signed Euclidean distance to Ω_*P*_ and the corresponding closest point on ${\hat{\gamma }}_{P}$ are calculated. The distributions of these distances and points respectively characterize how well the posterior distribution is centered around ${\hat{\gamma }}_{P}$ and the length of the curve which is covered by the posterior. To summarize the results from all the runs, 50% equal-tailed credible intervals for the two distributions were estimated for each run. Two of the 30 runs were rejected due to insufficient convergence of the Markov chains. For the remaining runs, the average interval-length along the curve is 0.148 (estimated as the Euclidean distance between the end-points, i.e., not taking the curvature into account), while the length perpendicular to the curve is 0.0127. For 22 of the 28 accepted runs, ${\hat{\gamma }}_{P}$ is within the perpendicular 50% credible interval, and it is within the 90% credible interval for all runs.

### 3.2. LH data

In this section, the estimation is performed on male and female LH data. The male data were collected in a clinical experiment described in detail in (23). A cohort of 18 healthy male test subjects participated in the study, where blood samples were drawn every 10 min over a time period of 21 h in four sessions. During three of the sessions, a dose of a selective GnRH-receptor antagonist, ganirelix, was administered 2 h into the session. In the remaining sessions, saline was administered instead. Three escalating doses of the GnRH-receptor antagonist were used, enabling analysis of the effect on the LH concentration when GnRH is suppressed to various extents. The female LH data were obtained through digitizing the representative LH profile of a woman in midluteal phase of the menstrual cycle depicted in **Figure 2** of (24).

We analyze these data in three ways. First, the curve *γ*_*P*_ is estimated from the 18-hour long male LH data sets without drug administration, and the female LH data set. Furthermore, we use a subset of one of the data sets used above to illustrate how the estimated time constants can influence the impulse estimation. Finally, the effect of the GnRH-receptor antagonist on the frequency and amplitude of the impulses is investigated along with the dependence of those on the age of the male test subjects. Here, the data points from hour 10 to hour 18 are used for the non-zero dose data, to ensure an approximately constant level of LH suppression by the antagonist.

Data from three individuals of age 68, 61 and 52 were excluded from the analysis in these experiments, as their LH profiles clearly deviated from the expected behaviour of sparse rapid bursts followed by slower elimination, which rendered the estimation unreliable. A figure comparing a typical included data set with an excluded one is provided in the **Supplementary Material**. In general, it is challenging to capture such dissimilar data sets with a single modeling framework. The higher ages of the excluded individuals are consistent with the experimentally established fact that the frequency of GnRH impulses increases, while their amplitudes decrease with age (25). Therefore, a higher sampling rate is required to distinguish between the individual pulsatile episodes and the LH signal.

To account for the unknown basal level of the hormone, the nadir value is subtracted from the data points of each data set. One outlier was removed from one data set as its implausibly low value interfered with establishing the basal level. There exist outlier detection methods that can be applied to hormonal data (26), but they are typically not adapted to the pulsatile nature of the signals, thus limiting their utility. In our case, the outlier was detected through visual inspection of the data. The distribution of all measurements for this individual, with the outlier highlighted, is shown in the **Supplementary Material**.

#### 3.2.1. Time constant estimation

The preceding results indicate that reliable estimates of *b_1_* and *b_2_* cannot be expected when there is significant uncertainty due to measurement noise or model mismatch. Since this is believed to be the case for the present data set, we will instead estimate the curve *γ*_*P*_ . It is, however, known from the underlying biochemistry that the true parameter values satisfy the inequalities (22)


(8) $$0.23{\text{ min}}^{-1}\leq b_{1}\leq 0.69{\text{ min}}^{-1},0.0087{\text{ min}}^{-1}\leq b_{2}\leq 0.014{\text{ min}}^{-1}.$$

**Figure 5:**
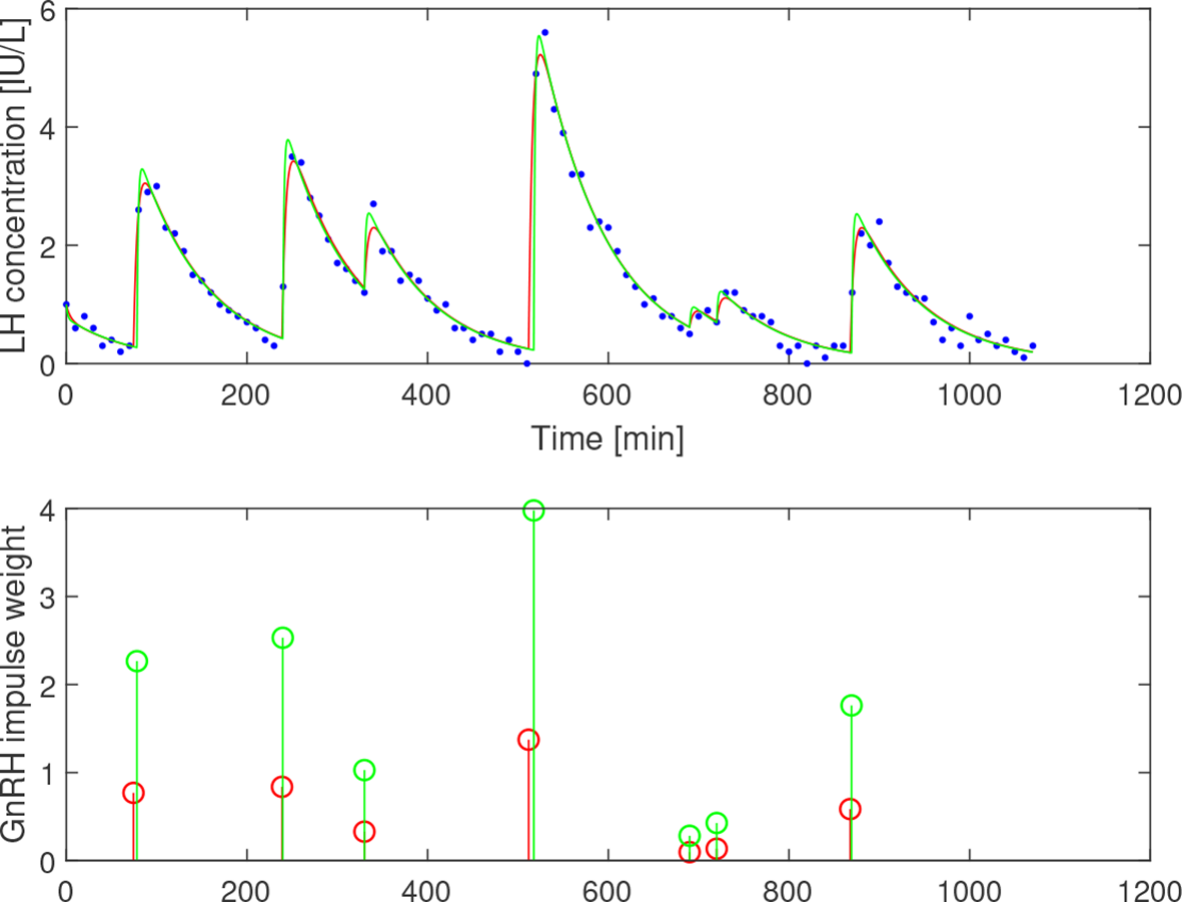
Estimation results assuming *b*_1_=0.23 (red) and *b*_1_=0.69 (green) based on LH data from a 23-year-old subject. Top: Simulated LH concentration and measured values (blue dots). Bottom: GnRH-impulses. *b_1_* mainly influences the weights of the impulses, while the firing times are largely unaffected.

To evaluate the proposed estimation approach, *γ*_*P*_ is estimated for the given range of *b*_1_, which produces a range of estimates ${\hat{b}}_{2}$ . These are then compared with the theoretical time-constant ranges, with the results displayed in **Figure 4**. There, 12 out of 16 curves are consistent with the theoretical parameter range, with no curve being 15% higher than the upper limit or 20% below the lower. The curve corresponding to female LH data is highlighted in the plot. No particular difference can be observed in either the measured concentrations or the estimation results between the male and midluteal female LH data. In **Figure 5**, the simulated LH concentration and the estimated GnRH-impulses for one individual of age 23 are shown. Here the output corresponding to the highest and lowest estimates in the *b_1_*-range are compared. The residual sum of squares is similar for the two cases; 2.71 and 2.59 (IU/L)^2^.

#### 3.2.2. Impulse–time constant relation

**Figure 6:**
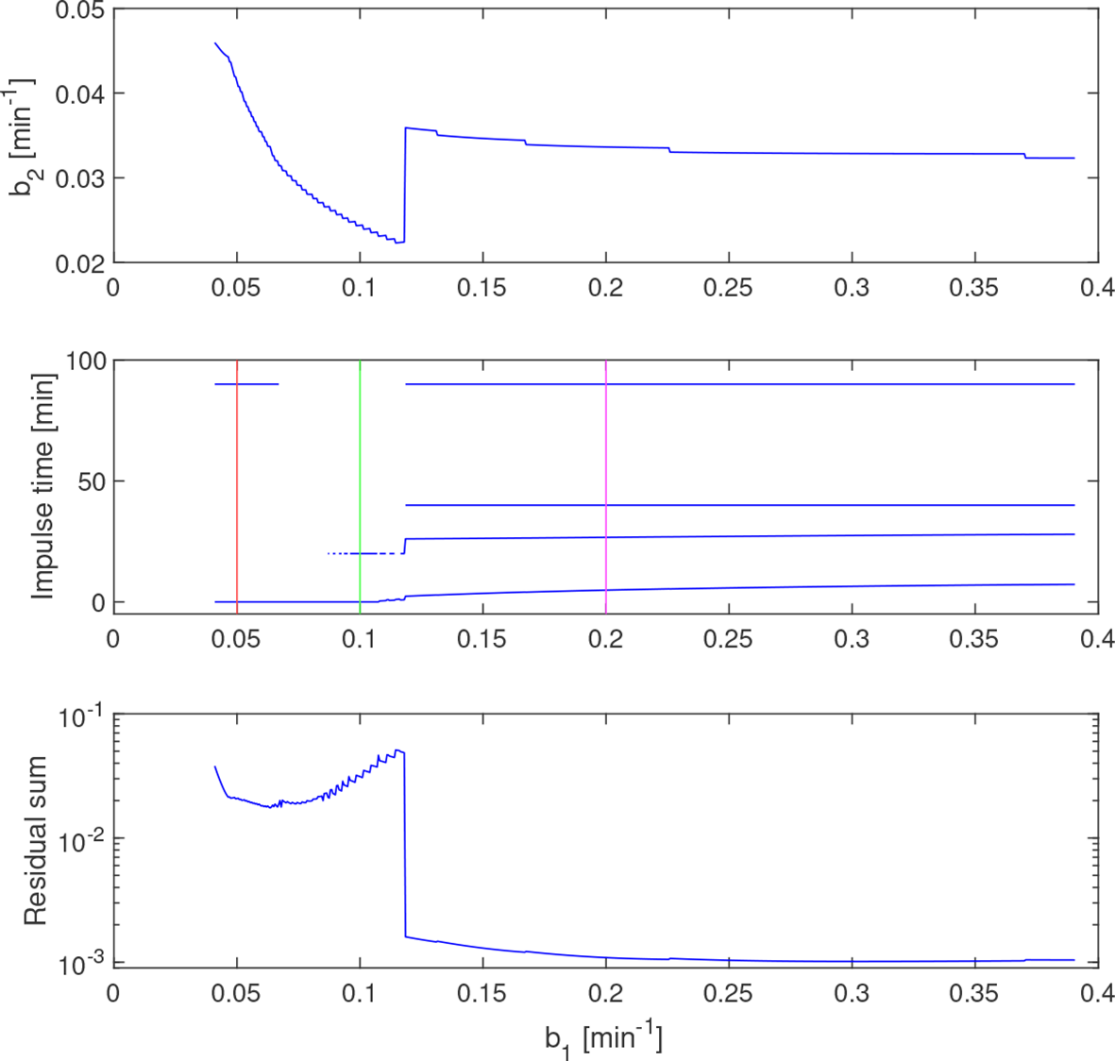
Estimated ${\hat{\gamma }}_{P}$ (top) and impulse times (middle) and residual sum of squares (bottom) along the curve ${\hat{\gamma }}_{P}$ . Vertical lines (red and green) correspond to the simulated concentrations in **Figure 7**. The discontinuity of ${\hat{\gamma }}_{P}$ coincides with the introduction of a fourth estimated impulse, which also reduces the residual sum.

**Figure 7:**
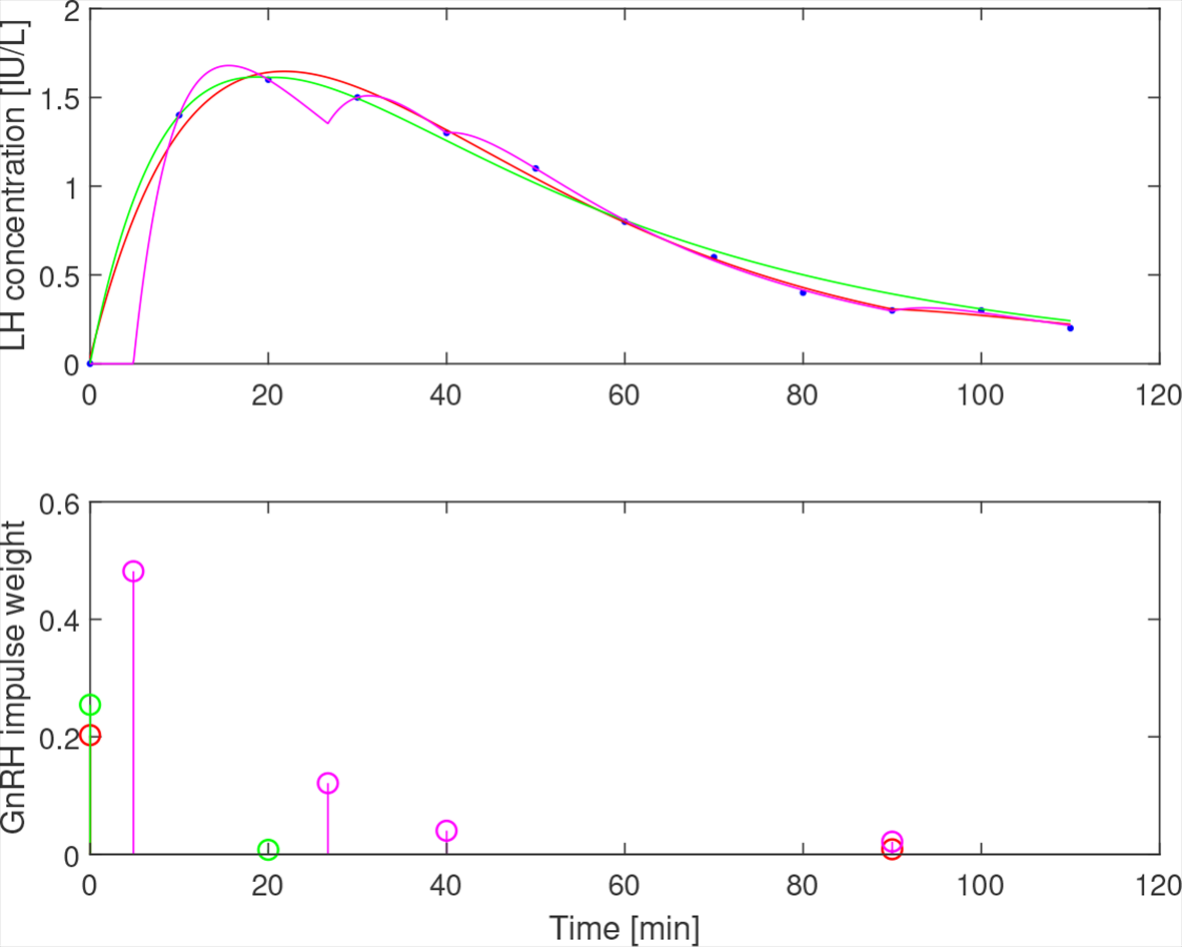
Simulated LH concentration (top) together with measured values (blue dots) and GnRH-impulses (bottom) from estimation assuming *b*_1_=0.05 (red), *b*_1_=0.1 (green) and *b*_1_=0.2 (magenta). Varying *b*_1_ results in qualitatively different impulse distributions, yet all three choices give a reasonable fit to the data.

The ${\hat{\gamma }}_{P}$ -curve provides a range of feasible solutions in terms of (*b*_1_,*b*_2_)-parameter pairs. However, the time constants also affect the estimated impulses. A subset of the LH-data from a 34-year-old individual is used here to demonstrate how variations in parameter values can lead to qualitatively different impulse distributions. The estimate ${\hat{\gamma }}_{P}$ is shown in **Figure 6** together with the estimated impulse locations and residual sum of squares along this curve. In the range of estimates, there are solutions with one, two, and four impulses, generally with a higher residual sum for the solutions with fewer impulses. The discontinuity of ${\hat{\gamma }}_{P}$ corresponds to a qualitative shift from two to four impulses. Simulated solutions for three values of *b_1_*, according to the markings in the middle plot in **Figure 6**, are displayed in **Figure 7**. One should note that the time constants used here are not within the theoretical bounds specified by (8).

#### 3.2.3. Effects of GnRH-receptor antagonist and age

The estimation is here performed on LH data sets affected by doses of 0.1, 0.3, and 1.0 mg/m^2^ of the GnRH-receptor antagonist ganirelix administered prior to the experiment. The weights and frequencies of the estimated impulses under medication are compared to those estimates without the drug. The latter estimation followed what is described in *Time constant estimation*, but with a fixed value b_1_=0.5 min^−1^ . Similar results are obtained with other values of *b_1_*, i.e., the sensitivity with respect to the parameter value is low, See **Supplementary Material**.

**Figure 8:**
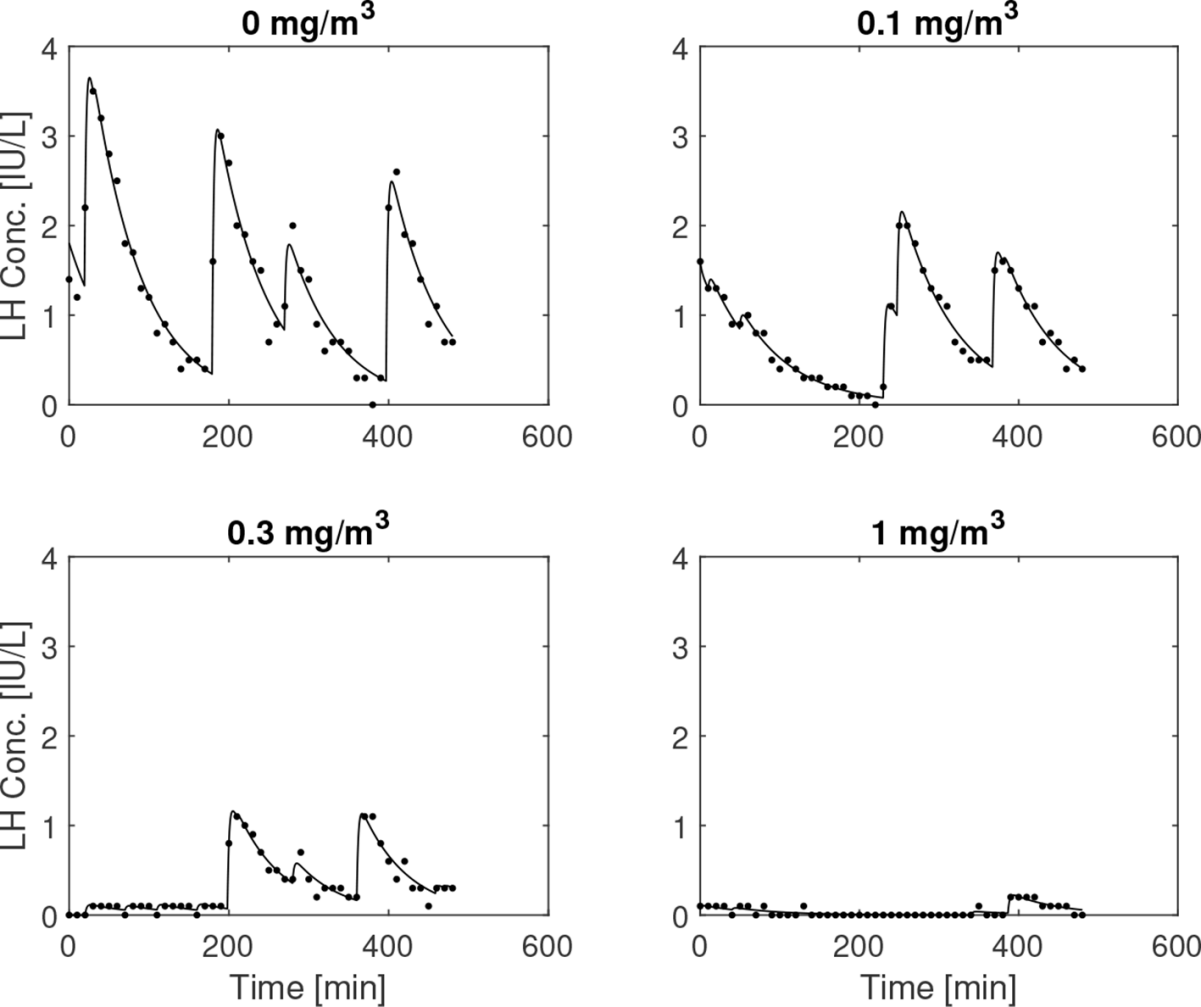
Simulated and measured LH concentration for escalating doses of ganirelix administered to a 24-year-old. The medication tends to lower both the amplitude and the frequency of the pulsatile episodes for this subject.

**Figure 9:**
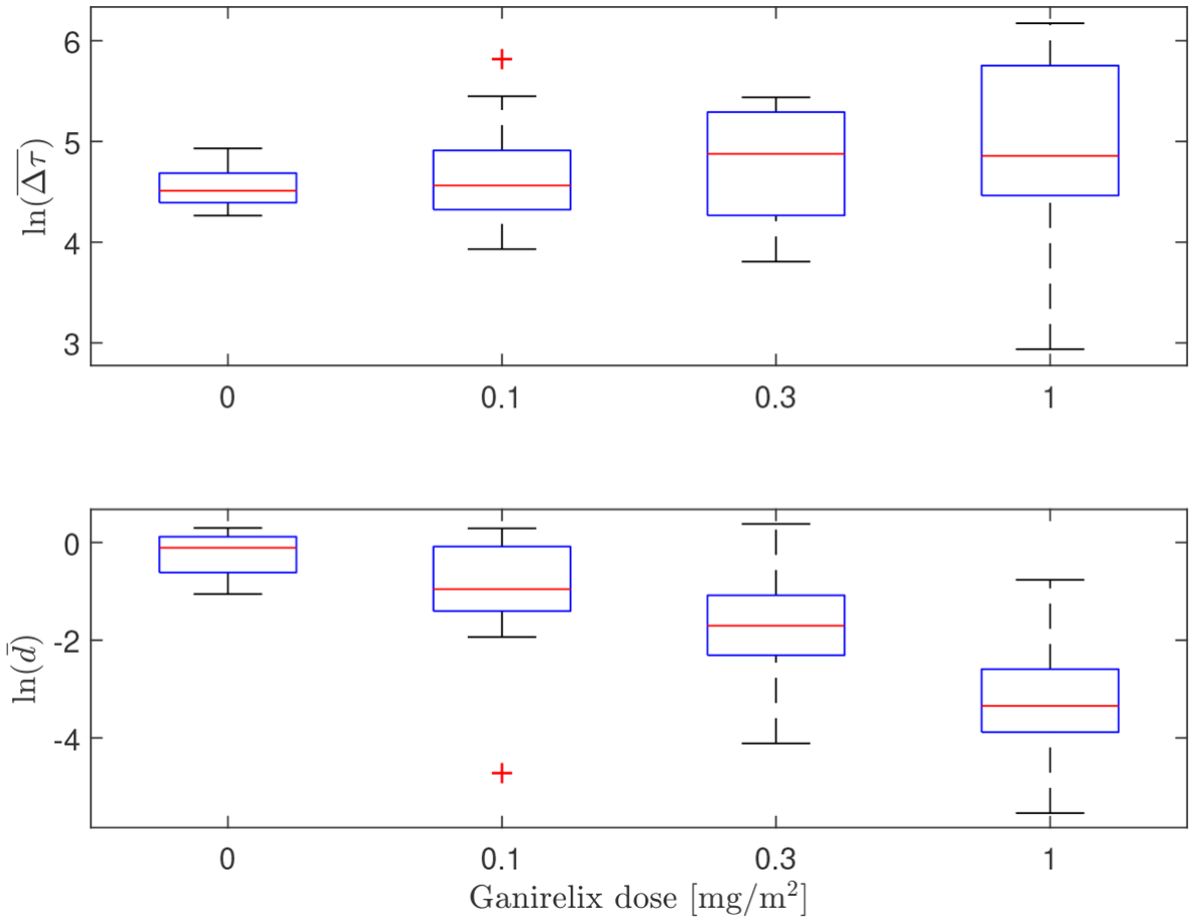
Box plot illustrating the distributions of estimated average impulse separation time (top) and impulse weights (bottom) for escalating doses of ganirelix administered to fifteen test subjects. Median values are indicated by the center line, the 25th and 75th percentiles are given by the box edges, whiskers show the most extreme values not considered outliers and outliers are marked by red crosses. Notice logarithmic scale on the ordinate axes.

In **Figure 8**, the influence of the GnRH-receptor antagonist on the LH concentration of one individual is illustrated. The trend of lower amplitude and frequency of the LH peaks as the dose is increased, which can be observed in this plot, is summarized for all individuals in **Table 1** along with the estimated values of ${\hat{b}}_{2}$ . In **Figure 9**, the distributions of the averages of the impulse time separations and weights are depicted, denoted as $\bar{\text{Δ}\tau }$ and $\overset{ˉ}{d}$ , respectively. One-way repeated measures analysis of variance was performed on (the logarithm of) these data, grouped by the administered doses of ganirelix. For $\bar{\text{Δ}\tau }$ , there is no statistically significant difference between the group means (*p*=.24), while the difference in $\overset{ˉ}{d}$ is significant (*p* <.001) *Post-hoc* analysis with a significance level of .05 and using Bonferroni correction shows that the highest ganirelix dose results in a *d*-mean that is significantly different from the rest of the groups and that the dose of 0.3 mg/m^2^ gives a mean that is significantly different from the unmedicated group. The short duration of the experiment in relation to the impulse frequency and uncertain estimates due to low signal-to-noise ratio are possible explanations for the inconclusive results for the effect of ganirelix on the impulse time separation.

**Table 1:** Parameter estimates with standard deviations for escalating doses of ganirelix.

Ganirelix dose [mg/m^3^]	${\hat{b}}_{2}$ [min1 ]	$\bar{\text{Δ}\tau }$ [min]	$\overset{ˉ}{d}$
0.0	0.0090 ± 0.0047	96.4 ± 19.6	0.85 ± 0.33
0.1	0.0089 ± 0.0045	120.1 ± 77.3	0.57 ± 0.40
0.3	0.0077 ± 0.0049	137.5 ± 70.2	0.32 ± 0.39
1.0	0.0056 ± 0.0041	203.6 ± 155.6	0.076 ± 0.12

**Figure 10:**
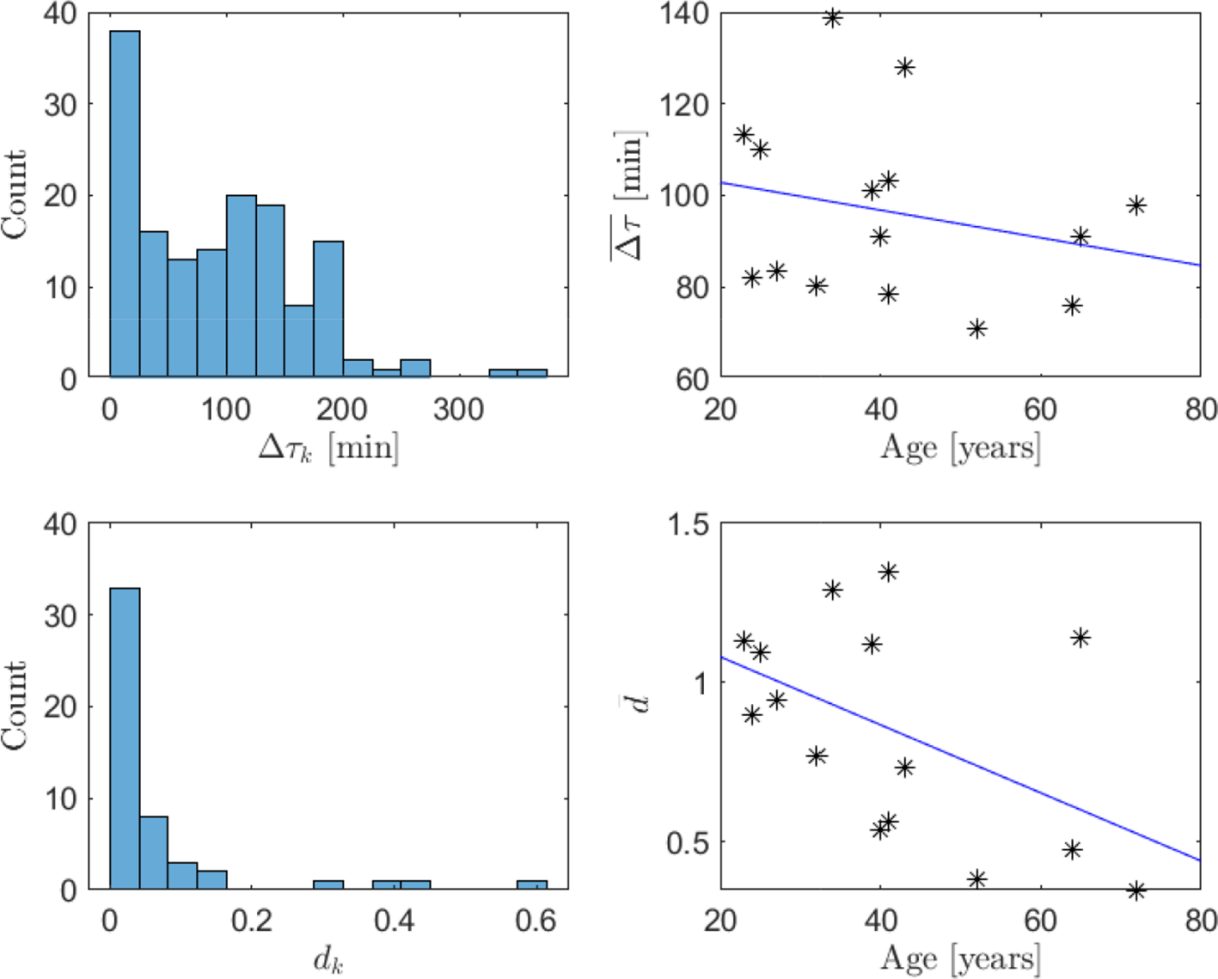
Histograms of impulse time separation (top left) and impulse weight (bottom left) for all individuals and average impulse time separation (top right) and impulse weight (bottom right) depending on age (both represented with asterisks), with fitted affine functions (in blue). The dependence of impulse weights on age is statistically significant (*p*=.029 ), whereas the dependence of time separation on age is not (*p*=.19).

Finally, the distribution of impulse weights and time separation for all subjects is shown in **Figure 10**, along with the age dependence of the average impulse time separation and weight, with fitted affine functions provided for reference. The weights display a unimodal distribution of *d*_*k*_ with a peak close to zero and a rapid decay for higher values, while the time separation shows less regularity. The relatively large number of instances of small impulse separation could indicate that impulses of non-instantaneous time-duration are present or, alternatively, nonlinearity of the hormone pharmacokinetics. It is expected that the impulse weights are reduced and the impulse frequency is increased with age (25). This behavior can be observed in the estimate averages where the time separation decreases with 0.30 min per year and the weights decrease with 0.011 per year. However, only the change in impulse weight is statistically significant with *p=*.029, while for time separation *p=*.19. The limitations in data-quality mentioned above presumably contribute to the observed uncertainty.

### 3.3. Cortisol data

The feasibility of the estimation method for other than LH hormone axes with pulsatile secretion is demonstrated in this section and exemplified by cortisol serum concentration data collected from three female adolescents, see (27). The subjects recruited in the study were 14–21 years old and included amenorrhoeic exercisers (AE), eumenorrhoeic exercisers (EE), and non-athletic controls (NE). The goal of the study was to demonstrate that higher levels of cortisol associated with physical exercise lead to lower LH secretion and might lie behind menstrual dysfunction in female athletes. Interestingly, a direct connection between the levels of LH and the secretion of cortisol in the female organism was observed in animal experiments, indicating that cortisol inhibits pituitary responsiveness to GnRH rather than suppresses hypothalamic GnRH release (28).

The data points for modeling are obtained through digitizing the figures provided in (27) as typical cortisol profile examples for the corresponding subject group. For each subject, blood samples were drawn every 10 min during an eight-hour overnight period, with an assessment for cortisol applied to every second sample. We refer to (27) for more details on the experimental protocol.

Notice that the sampling frequency for the cortisol data set is only half of what is considered in *LH data*. The half-life time of cortisol is approximately three times longer than that of LH. The regulation of cortisol involves two more hormones, namely corticotropin releasing hormone (CRH) and adrenocorticotropic hormone (ACTH). The pharmacokinetics of CRH cannot be captured in the data due to its fast half-life time of 4 min. Even for ACTH, the sampling rate of 20 min is slow as the half-life time is known to vary in the range of 10–30 min. Undersampling of a signal is known to lead to aliasing artifacts, i.e., spurious components of lower frequency.

The data were analyzed in (27) by means of AutoDecon yielding estimates of cortisol half-life time and the parameters of the secretion episodes. For the half-life time, the reported estimates are 60.7 min, 47.3 min, and 49.8 min for the AE, EE, and NE cohorts, respectively.

**Figure 11:**
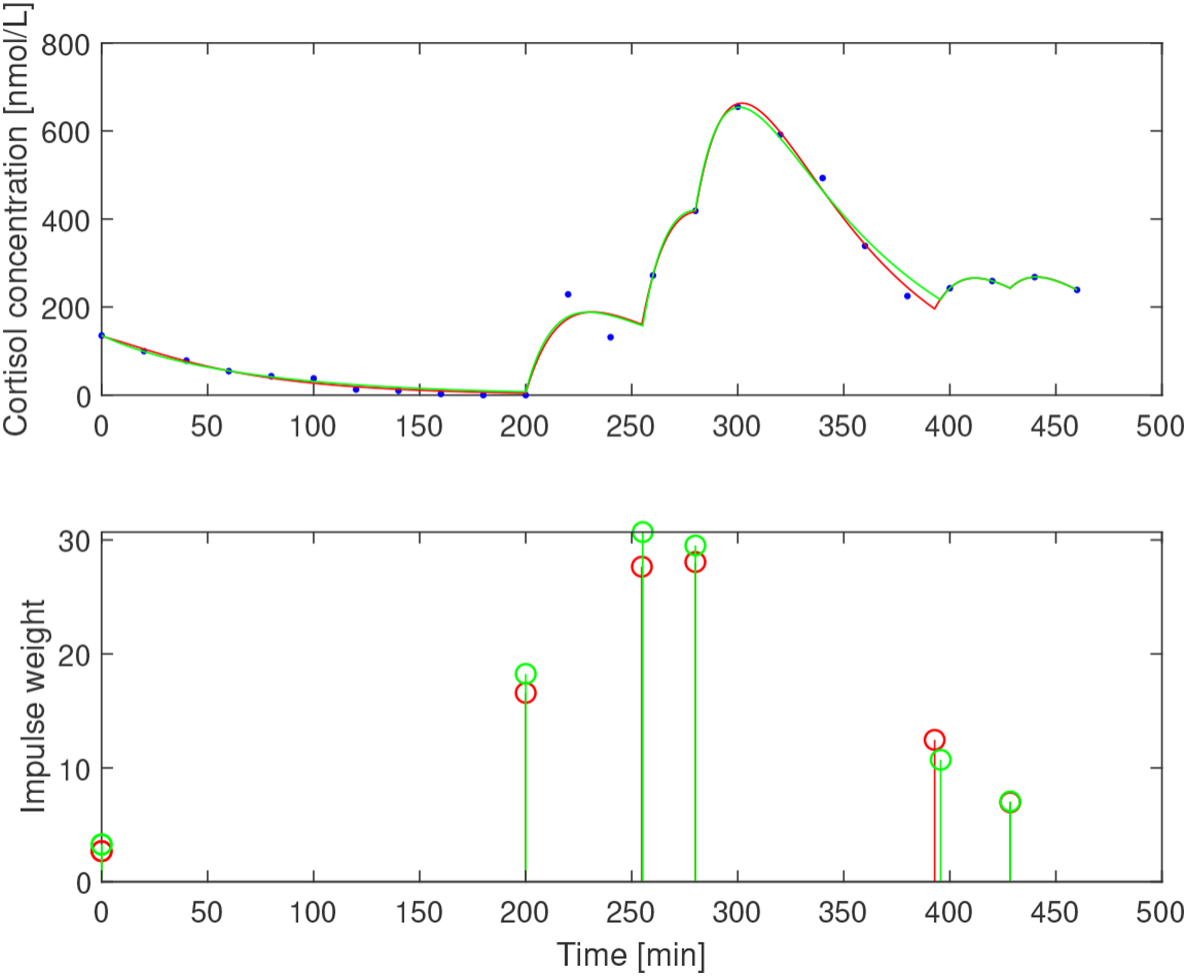
Simulated cortisol concentration (top) together with measured values (blue dots) and impulse estimates (bottom) from estimation assuming *b*_1_=0.05 min^−1^ (red) and *b*_1_=0.065 min^−1^ (green) for amenorrheic exerciser.

**Figure 12:**
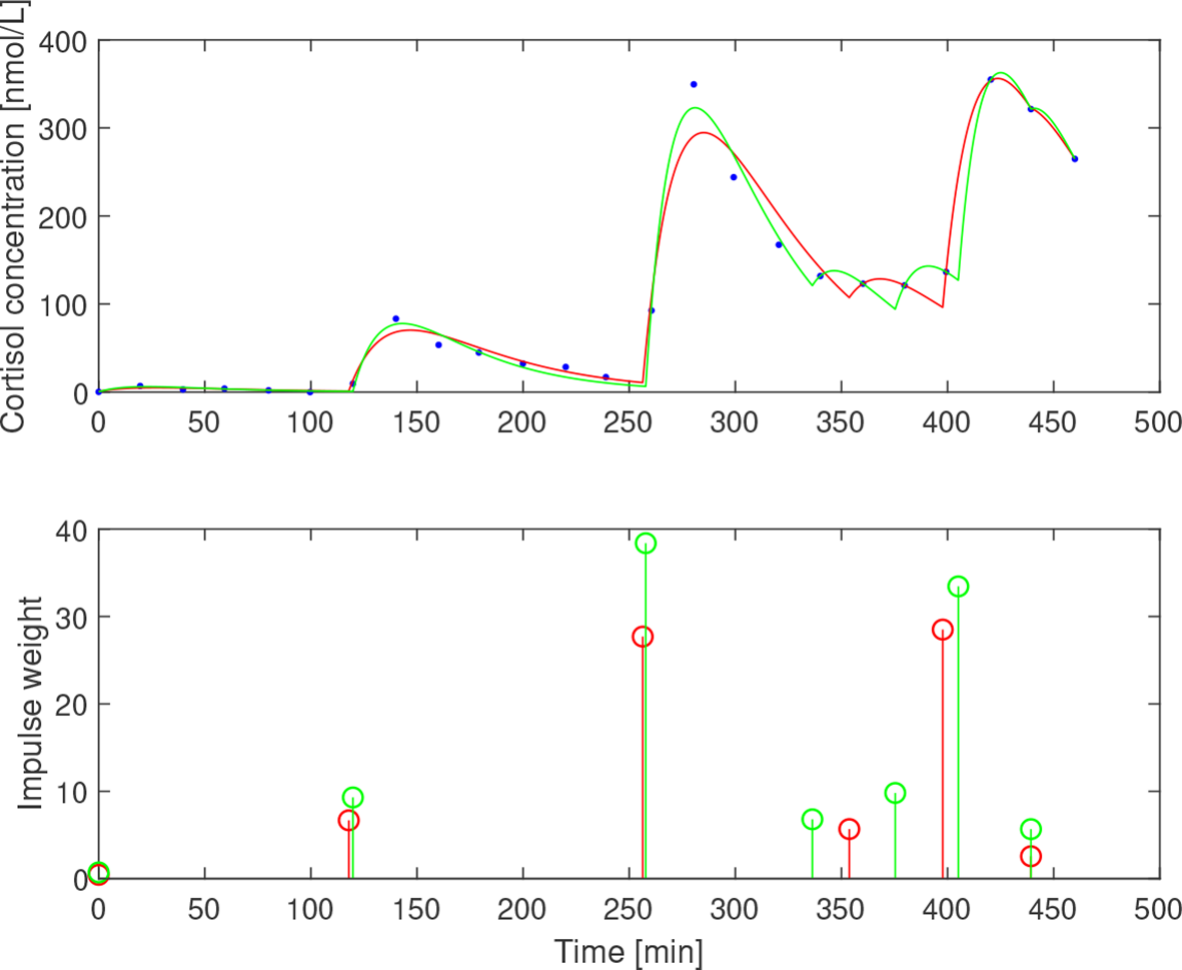
Simulated cortisol concentration (top) together with measured values (blue dots) and impulse estimates (bottom) from estimation assuming *b*_1_=0.05 min^−1^ (red) and *b*_1_=0.065 min^−1^ (green) for eumenorrheic exerciser.

**Figure 13:**
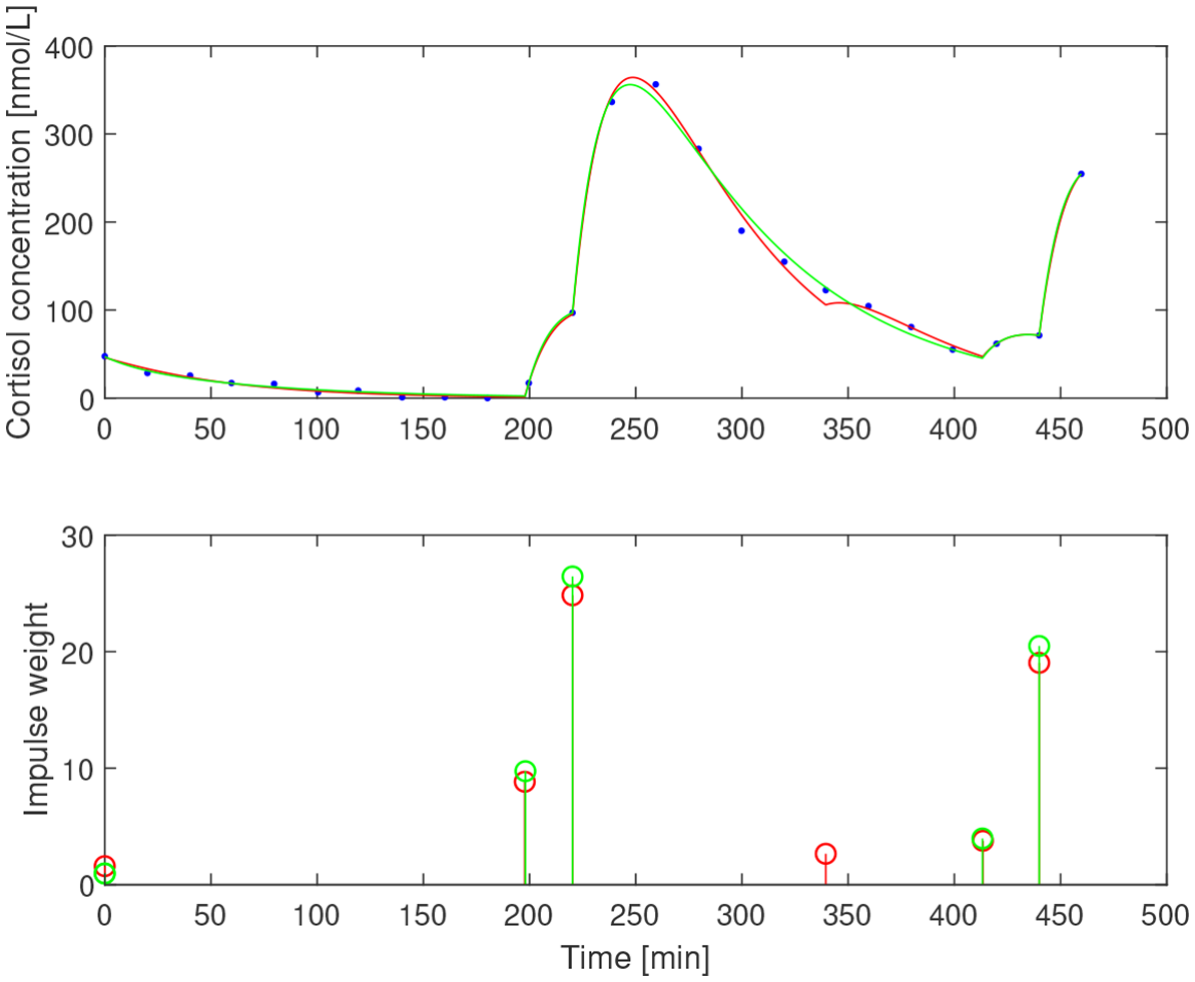
Simulated cortisol concentration (top) together with measured values (blue dots) and impulse estimates (bottom) from estimation assuming *b*_1_=0.05 min^−1^ (red) and *b*_1_=0.065 min^−1^ (green) for non-exerciser.

After subtracting the basal concentration assumed to coincide with the minimal measured concentration, we estimated ${\hat{\gamma }}_{P}$ for the three subjects. The impulse times and weights were then estimated for two values of the elimination rate *b*_1_=0.05 min^−1^ and *b*_1_=0.065 min^−1^ that correspond to half-life of ACTH of 13.9 min and 10.7 min, respectively. The resulting impulses and concentration profiles are displayed in **Figures 11**–**13**. The corresponding half-life estimates for cortisol are 38.1 min and 48.4 min (AE), 31.1 min and 26.4 min (EE), and 37.6 min and 50.0 min (NE). Compared to the population-wide estimates in (27), these estimates are within the same range and feasible from a biochemical point of view, despite the already mentioned undersampling issue.

**Figure 14:**
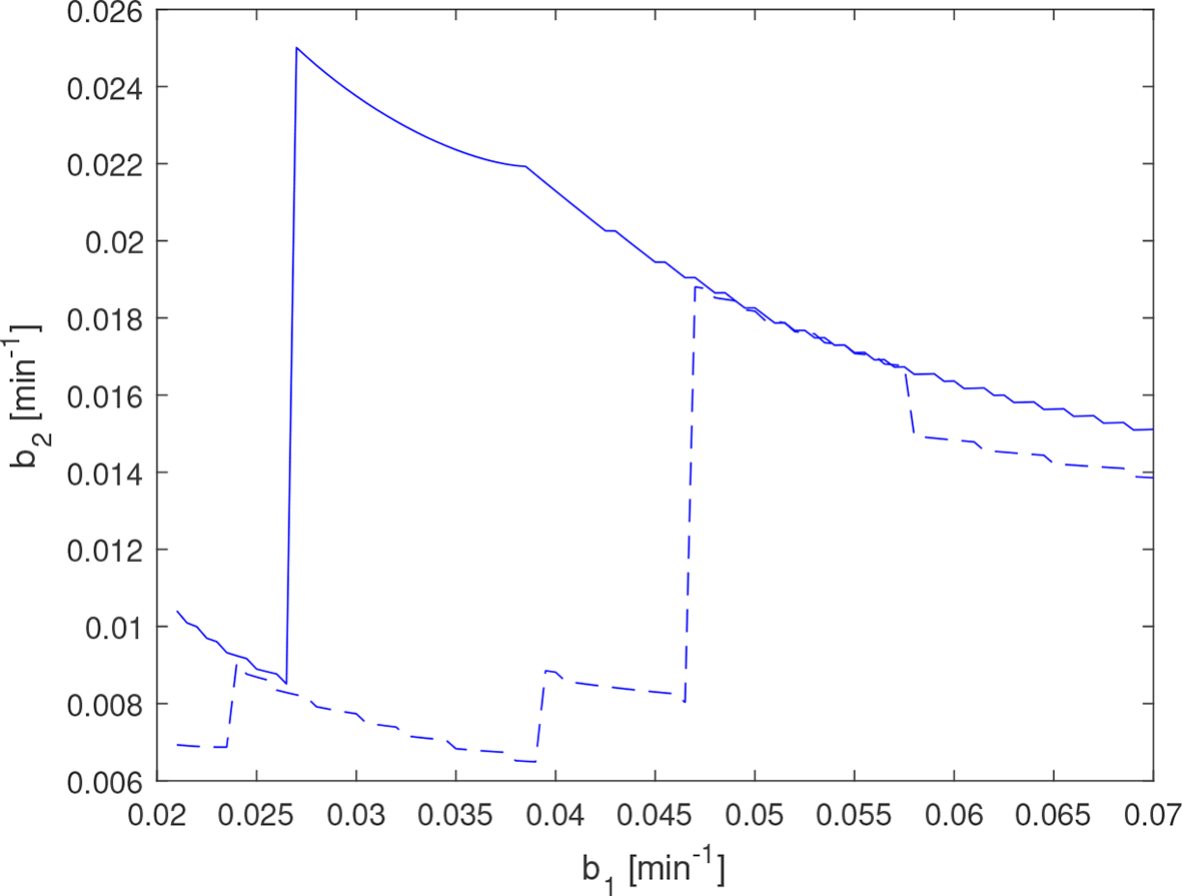
${\hat{\gamma }}_{P}$ for amenorrheic exerciser estimated with complete data (dashed line) and with the 220 min measurement in the data set (see **Figure 11**) removed (solid line). The discontinuity at *b*_1_=0.047 min^−1^ for the dashed line corresponds to a reduction in the number of estimated impulses for *b*_1_≤0.047min^−1^ , which in this instance results in a significantly worse model fit.

The time series estimation performance deteriorates for the data for AE and EE subjects when a lower value of *b_1_* is chosen, as the corresponding estimate of *b_2_* then tends to be too low and results in a bad fit to the data. For the AE subject, this problem is mitigated by removing the data point at 220 min, which indicates a sensitivity of the estimate to outliers on undersampled data, as expected. In **Figure 14**, the estimates of ${\hat{\gamma }}_{P}$ evaluated with and without this outlier point are depicted for comparison. In the region 0.027 min^−1^≤*b*_1_≤0.047 min^−1^ , the higher value of *b_2_* improves the model fit significantly, resulting in approximately 30 times lower residual error.

## 4 Discussion

We have presented a model-based method for estimating the pulsatile bursts and elimination rates in biochemical data, e.g., endocrine data. The properties of the method have been examined using synthetic data, and its utility has been demonstrated on clinical male and female LH as well as cortisol data.

In the experiments with synthetic data, it can be seen that the estimated curve ${\hat{\gamma }}_{P}$ coincides with the region of maximum-likelihood obtained from the AM algorithm. The least-squares algorithm that is employed to derive the curve is however, computationally cheaper compared to the MCMC estimation (see *Functional relation between the elimination rates*). It is also shown that additive noise renders the estimation problem ill-posed, as the parameter combinations along ${\hat{\gamma }}_{P}$ produce similar posterior probability densities. This is an instance of the notoriously difficult problem of estimating the parameters of sums of exponentials, but the particular functional relationship it results in the problem at hand seems to have been overlooked in the past. One reason for this is that the model parameters are often characterized by their point estimates (with or without uncertainties) or confidence intervals, while capturing a functional relationship requires an analysis of the covariance between the parameters or a visualization of the marginal probability density to be observed. However, as the noise level decreases, the problem becomes more well-posed. Furthermore, even with significant noise, the marginal posterior distribution has a bounded support that does not cover the full range of the ${\hat{\gamma }}_{P}$ -curve. It therefore appears that true parameter values and other feasible parameter values are necessarily close to the ${\hat{\gamma }}_{P}$ -curve, but being close to the curve is not a sufficient condition for a parameter set to be feasible. Finding a sufficient feasibility condition based on the least-squares formulation is a relevant question for further research.

## Data availability statement

The data analyzed in this study is subject to the following licenses/restrictions: The modeling part of this study makes use of clinical data described in https://doi.org/10.1152/ajpregu.00138.2005. The authors do not own the data and therefore cannot make them available. Requests to access these datasets should be directed to veldhuis.johannes@mayo.edu. Code for the estimation algorithms is publicly available and can be downloaded at https://github.com/HRunvik/Impulsive-Time-Series-Modeling.

## Ethics statement

The studies involving human participants were reviewed and approved by The Mayo Clinic Institutional Review Board, Mayo Clinic. The patients/participants provided their written informed consent to participate in this study.

## Author contributions

HR conceived and implemented the least-squares estimation algorithm, implemented the MCMC algorithm, and performed the experiments, under the guidance of AM. HR prepared the manuscript, with input from AM. Both authors revised the manuscript. All authors contributed to the article and approved the submitted version.

## Funding

This work is funded in part by the PhD program at the Centre for Interdisciplinary Mathematics, Uppsala University, Sweden and the Swedish Research Council Grant 2019-04451 for the project “Synchronization and entrainment in the impulsive Goodwins oscillator.”

## Acknowledgments

The authors gratefully acknowledge the contribution of clinical data for this study by Prof. Johannes D. Veldhuis.

## Conflict of interest

The authors declare that the research was conducted in the absence of any commercial or financial relationships that could be construed as a potential conflict of interest.

## Publisher’s note

All claims expressed in this article are solely those of the authors and do not necessarily represent those of their affiliated organizations, or those of the publisher, the editors and the reviewers. Any product that may be evaluated in this article, or claim that may be made by its manufacturer, is not guaranteed or endorsed by the publisher.
