# Supplementary material for: Impulsive time series modeling with application to luteinizing hormone data
Source: Front Endocrinol (Lausanne). 2022 Nov 1;13:957993. doi: 10.3389/fendo.2022.957993 (PMC9664167; doi:10.3389/fendo.2022.957993)
Supplement: Supplementary file 1 [file DataSheet_1.pdf]

Supplementary Material

1 INPUT SEQUENCE AND PARAMETER ESTIMATION ALGORITHM

This section contains further details on the estimation algorithm in Runvik and Medvedev (2022). Two cases are considered; estimation in the absence of noise and estimation under uncertainty.

1.1 Noise-free estimation

In the noise-free case, the estimation utilizes how the solutions to the optimization problem (5) divide the b_1-b_2-parameter space into distinct regions. In this setup we allow negative impulse weight estimates, and use how the signs of the impulses depend on the parameter values to navigate to the true parameter values b_1^*, b_2^*. The following proposition provides an initial division of the space.

PROPOSITION 1. *Runvik and Medvedev (2022) Consider LS problem* (5) *with the initial condition* $x_2(t_1) = 0$*. Assume that the noise-free measurements* Y *are produced by* (2) *and that* $b_1 < b_2$, $b_1^* < b_2^*$*. Let* $S = \{k \in \{1, \ldots, K\} \mid d_k^* = 0\}$*. If* $b_1 + b_2 > b_1^* + b_2^*$ *and* $b_1 > b_1^*$*, then* $d_k > 0$ *for* $k = 1, \ldots K$*. If* $b_1 + b_2 < b_1^* + b_2^*$ *and* $b_1 < b_1^*$*, then* $d_k < 0$ *for* $k \in S$*.*

A further refinement of this division is derived by considering the estimated impulse weights in a simplified case with three measurements of the response of a single impulse. Each such triplet gives rise to a curve in the b_1-b_2-space, defining the boundary between regions of positive and negative impulse weight estimates. By assuming that properties of this type of curve also hold in the general setting (which appears to be true in numerical simulations), and assuming sufficient sparsity of the impulses in relation to the sampling rate, the maximum and minimum of all possible curves generated by any triplet of measurements define two curves in the parameter space, respectively representing the boundaries of the regions with positive and negative impulse weights. For the positive region, this is the curve γ_P that is sketched in Fig. 1. Importantly, the boundary of the negative region meet γ_P only the point (b_1^*, b_2^*), which enables the determination of these parameters by locating the point where the boundaries meet.

1.2 Estimation under uncertainty

When additive measurement noise is present, the parameter space is no longer divided into the well-defined regions outlined above, instead impulses with both positive and negative weights would be typically be present in most of the parameter space. To robustify the estimation, a non-negativity constraint on the impulses is therefore added in the optimization. This produces the alternative characterization of γ_P based on zero loss given in Section 2.1.1 and motivates the estimation procedure using (6) and (7).

2 LEAST SQUARES IMPLEMENTATION

The methods used to solve optimization problem (6), and thus enabling the estimation of γ_P, are presented here. It can be seen in numerical experiments that this problem generally tends to be non-convex, but with only few (typically one or two) local minima located in a small interval. Assuming that no prior information is available to determine a good starting guess for b_2, we use a refined gridding strategy for this problem in the experiments with synthetic data in Section 3.1. Algorithm 1 is then modified so that $\tilde{b}_2$ is first calculated over a coarse grid of b_1 and b_2, giving estimates $\tilde{b}_2(b_{1,\mathrm{c}}^{(n)}), n = 1, \ldots N_\mathrm{c}$. Then a finer grid over b_1 is used

where, for each $b_{1,\text{f}}^{(n)}, n = 1, \ldots N_\text{f}$, a grid in the b_2 direction over the range $[\tilde{b}_2(\tilde{b}_1^{(n)}) - \delta, \tilde{b}_2(\tilde{b}_1^{(n)}) + \delta]$, where $\tilde{b}_1^{(n)}$ is the point of the coarse grid closest to $b_{1,\text{f}}^{(n)}$ and δ is a positive parameter. In both grids, a finite difference approximation over the points in the b_2-grid is utilized to approximate the derivative of the residual sum. The distance between the points of the coarse grid is set to 0.02 (in both directions); the corresponding value for the fine grid set to 0.002. $\delta = 0.06$ is furthermore chosen.

In the estimation from clinical data, where the LH level is not reduced by medication in Section 3.2, gridding is used without the refinement described above. In Section 3.2.1, the distance between the points of the grid is 0.002 in the b_1-direction (with the range defined by (8)) and 5×10^{-4} in the b_2-direction (in the range $[0.0015, 0.04]$) while 5×10^{-4} is used in both directions in Section 3.2.2. In the estimation with cortisol data, the distance between grid points is 5×10^{-4} in the b_1-direction and 2×10^{-4} in the b_2-direction. The parameter $d_{\min}$ is set to 5% of the maximal estimated impulse weight in Section 3.2.1, 3.2.3 and 3.3 and 2% is Section 3.2.2 (the lower value of is used as the noise level appears lower in the limited data-set considered in that experiment), while d_Π is set to 5% of the mean estimated impulse weight and $\Pi = K/2$ in all experiments.

Finally, for the data sets where the LH level is reduced by the GnRH-receptor antagonist, the estimated value of b_2 for each individual in the unmedicated case is used for initializing an interior point optimization with the Matlab function `Fmincon`, where the derivative in the objective function again is approximated with a finite difference. This strategy makes the estimation more robust compared to the gridding in this instance, when the medication decreases the signal-to-noise ratio.

3 ADAPTIVE METROPOLIS IMPLEMENTATION

3.1 Empirical covariance

In the AM algorithm, a Gaussian zero-mean proposal distribution is used with a covariance which depends on the iteration index i and the states of the Markov chain (i.e. the parameter vector) Θ_i according to

$$C_i = \begin{cases} c_0 & \text{if } i \leq i_0, \\ s_d \left(c(\Theta_1, \ldots \Theta_i) + \epsilon I \right) & \text{otherwise,} \end{cases}$$

where c_0 is a fixed covariance matrix, i_0 is a positive integer, s_d and ϵ are positive scaling parameters, I denotes the identity matrix and $c(\Theta_1, \ldots \Theta_i)$ is the empirical covariance matrix

$$c(\Theta_1, \ldots \Theta_i) = \frac{1}{i} \sum_{k=1}^{i} \Theta_k \Theta_k^\intercal - (i+1) \bar{\Theta}_i \bar{\Theta}_i^\intercal,$$

where the bar denotes the mean of the states over indices $1, \ldots i$. In the present work, the parameter values $c_0 = 0.1I$, $i_0 = 2$, $s_d = 0.02$ and $\epsilon = 0.01 s_d$ were chosen.

3.2 Superfluous impulse removal

The method for identifying and removing superfluous impulses from the posterior samples of the AM algorithm is given here. For each sample, Algorithm 1 is performed. Here N is the assumed number of impulses, $\delta > 0$ is a time relaxation parameter and $d_{\min} > 0$ is the impulse weight threshold. The parameter values $\delta = 0.1$, $d_{\min} = 0.1$ were used in the numerical experiments.

Algorithm 1 Superfluous impulse removal

for $n \in \{1, \dots, N\}$ **do**
    if $n < N$ **and** $\exists\, t_i, t_{i+1}$ s.t. $t_i < \tau_n < \tau_{n+1} < t_{i+1} + \delta$ **then**
        Merge impulses $n, n+1$ according to Algorithm 1 in Mattsson and Medvedev (2015)
    else
        if $d_i < d_{\min}$ **or** $\tau_n + \delta > t_{\text{end}}$ **then**
            Remove impulse n
        end if
    end if
end for

3.3 Gelman-Rubin statistic

Convergence of the AM algorithm is verified using the Gelman-Rubin statistic (Gelman and Rubin, 1992). This verification is based on running multiple Markov chains for the same estimation, and then comparing the within-chain and between-chain variability of the estimates. In particular, unbiased estimates of the variances of each parameter are calculated in two ways; using the variance between chains and through averaging over all chains. The square root of the ratio between these estimates produces the statistic R, which approaches 1 as the chains converge. In our work, we use the mean value of R over all estimated parameters to verify convergence.

4 SYNTHETIC DATA EXPERIMENT

The probability distributions and the fixed parameters used to generate the synthetic data used in Section 3.1 are summarized in Table S1. For both the least squares and the AM estimation, b_1 and b_2 were restricted to the ranges

$$\frac{b_2^*}{2} \le b_2 \le \frac{b_2^* + b_1^*}{2}, \quad \frac{b_2^* + b_1^*}{2} < b_1 \le \frac{3b_1^*}{2}.$$

Constraints restricting the impulse times to be within the time horizon and the impulse weights to satisfy $0 \le d_i \le 8$ (i.e. twice the maximal amplitude allowed for the generating model) were also used for the AM algorithm. Five impulses were assumed in the AM algorithm, see the discussion in Section 2.2 and 3.

Table S1. Experiment parameters for synthetic data generation. $U_{[\cdot,\cdot]}$ is the uniform distribution, $\Delta\tau_i$ denotes the time separation between the impulses and $t_{\text{end}} - \tau_{\text{end}}$ defines the time between the last impulse and the end of the data set.

b_2^*	$U_{[0.4,1.4]}$
$b_1^* - b_2^*$	$U_{[0.3,1.3]}$
d_i^*	$U_{[0.4,4]}$
$\Delta\tau_i$	$U_{[1.5,5]}$
$t_{\text{end}} - \tau_{\text{end}}$	5
Number of runs	30
Noise std	0.004
Sampling rate	0.5
Number of impulses	3

5 OUTLIERS AND DEVIATING HORMONE PROFILES

Further information on measurements and data sets that were removed in the estimation experiments in Section 3.2 are given in this section.

In Fig. S1, typical LH profiles for removed and included subjects are illustrated.

In Fig. S2 the distribution of LH measurements from a 32-year-old is displayed. The highlighted outlier was removed from this data set as it is clearly outside the distribution of the remaining measurements for this individual.

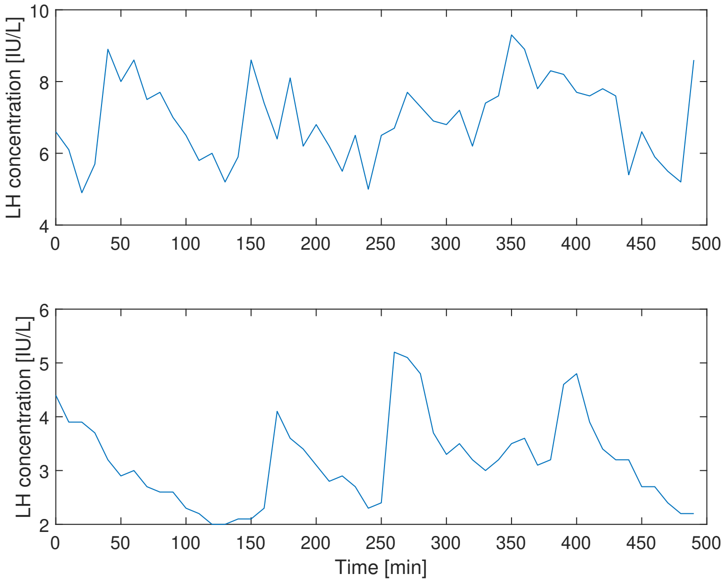

Figure S1. Subsets of the LH concentration profiles for a 68-year-old (top) and 24-year-old (bottom). The dataset corresponding to the upper plot was removed due to the deviating behaviour.

6 SENSITIVITY ASSESSMENT FOR GNRH-RECEPTOR ANTAGONIST AND AGE EFFECTS

In Section 3.2.3, the value of b_1 is set to 0.5. To assess the sensitivity with respect to this choice, we compare the results when $b_1 = 0.35$, $b_1 = 0.5$ and $b_1 = 0.65$ in this Section. Estimation results are summarized in Table S2. Some differences can be observed, but the results appear not to be particularly sensitive to the choice of b_1 overall.

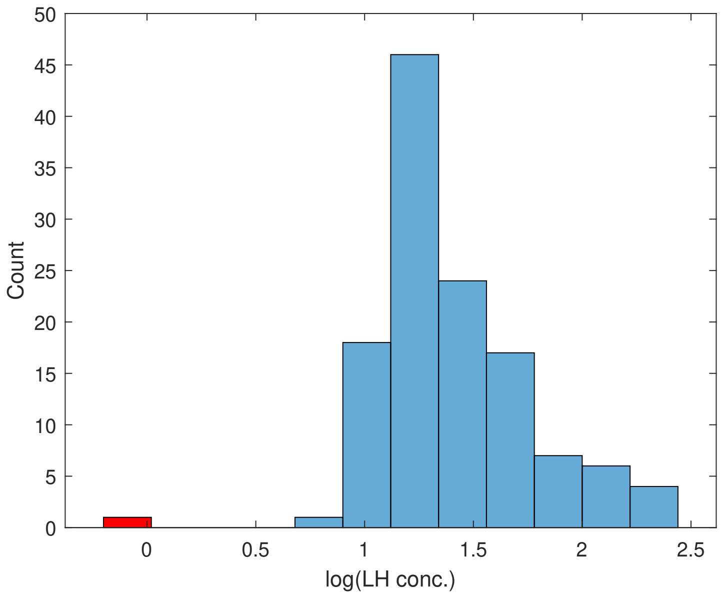

Figure S2. Logarithm of LH concentration measurements from 32-year-old subject. The outlier marked in red was removed from the data set.

Table S2. Estimation results for the experiments described in Section 3.2.3 for different values of b_1. The first two rows contain the results of repeated one way analysis of variance of the average time separation between impulses and the average impulse weight with respect to different doses of Ganirelix. The last two rows contain the results from fitting affine curves to the same estimates as functions of the ages of the subjects. The p-values there are with respect to the null hypothesis of no age dependence, i.e. a zero slope.

	$b_1 = 0.35\ \mathrm{min}^{-1}$	$b_1 = 0.5\ \mathrm{min}^{-1}$	$b_1 = 0.65\ \mathrm{min}^{-1}$
Significant Ganirelix effect on $\overline{\Delta\tau}$	Yes ($p = 0.046$)	No ($p = 0.24$)	No ($p = 0.23$)
Significant Ganirelix effect on $\bar{d}$	Yes ($p < .001$)	Yes ($p < .001$)	Yes ($p < .001$)
$\overline{\Delta\tau}$ age slope [min/yr]	-0.0084 ($p = .095$)	-0.011 ($p = .30$)	-0.014 ($p = .28$)
$\bar{d}$ age slope [min/yr]	-0.50 ($p = .022$)	-0.30 ($p = .029$)	-0.18 ($p = .028$)
